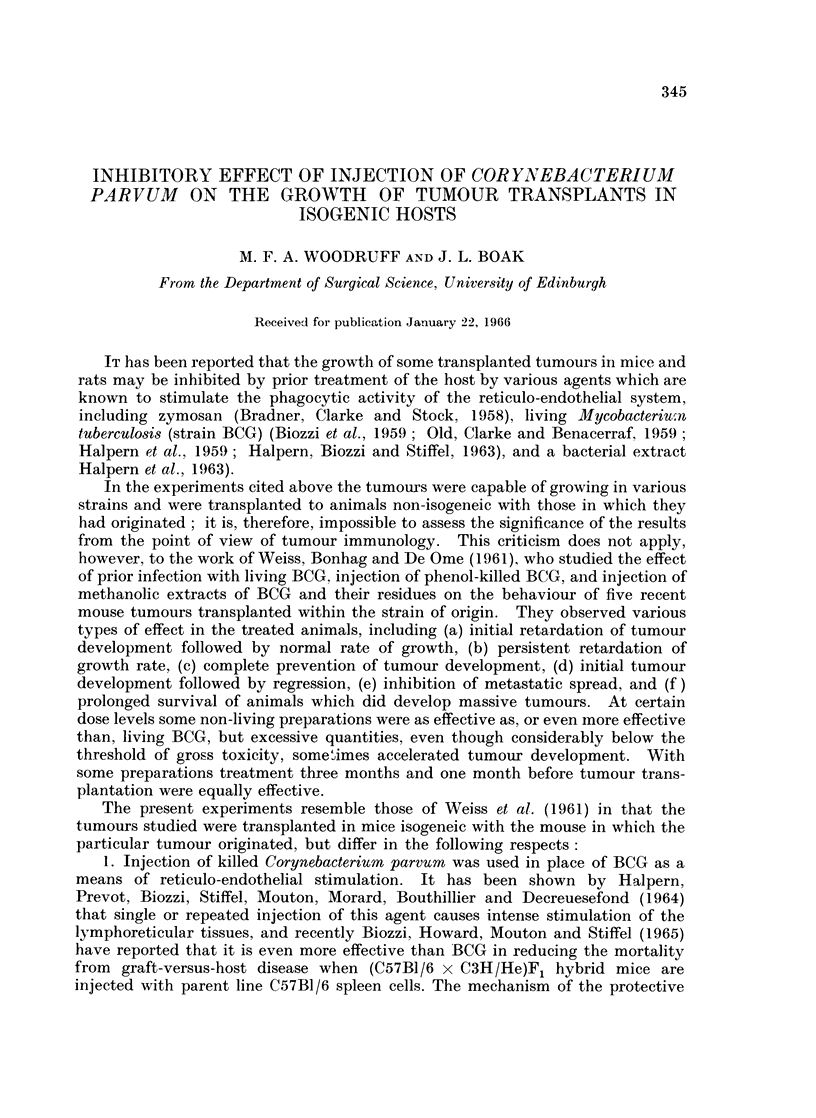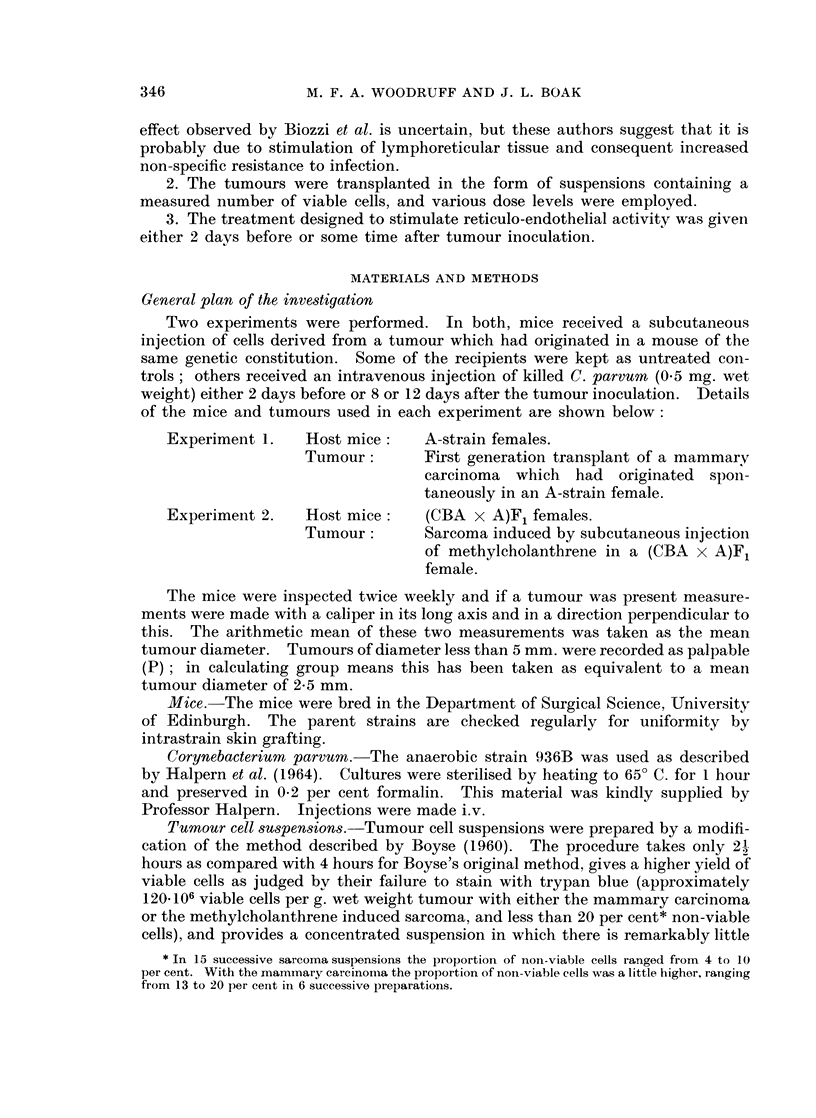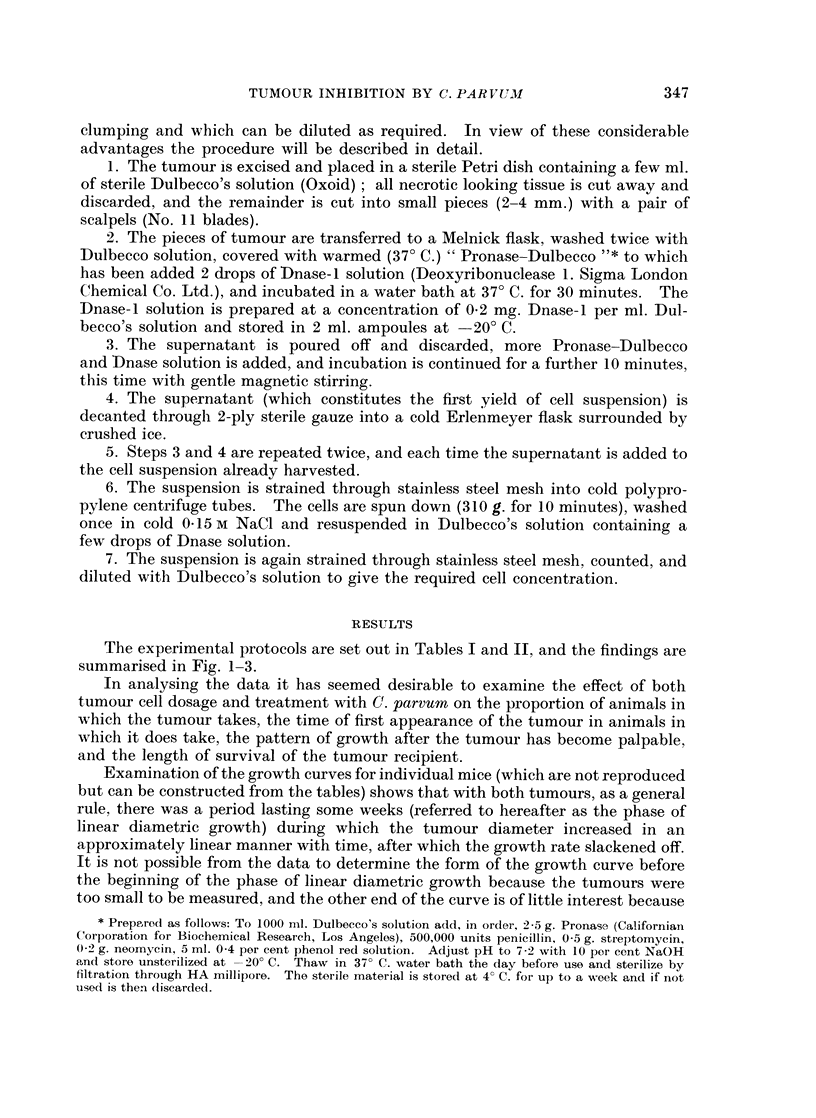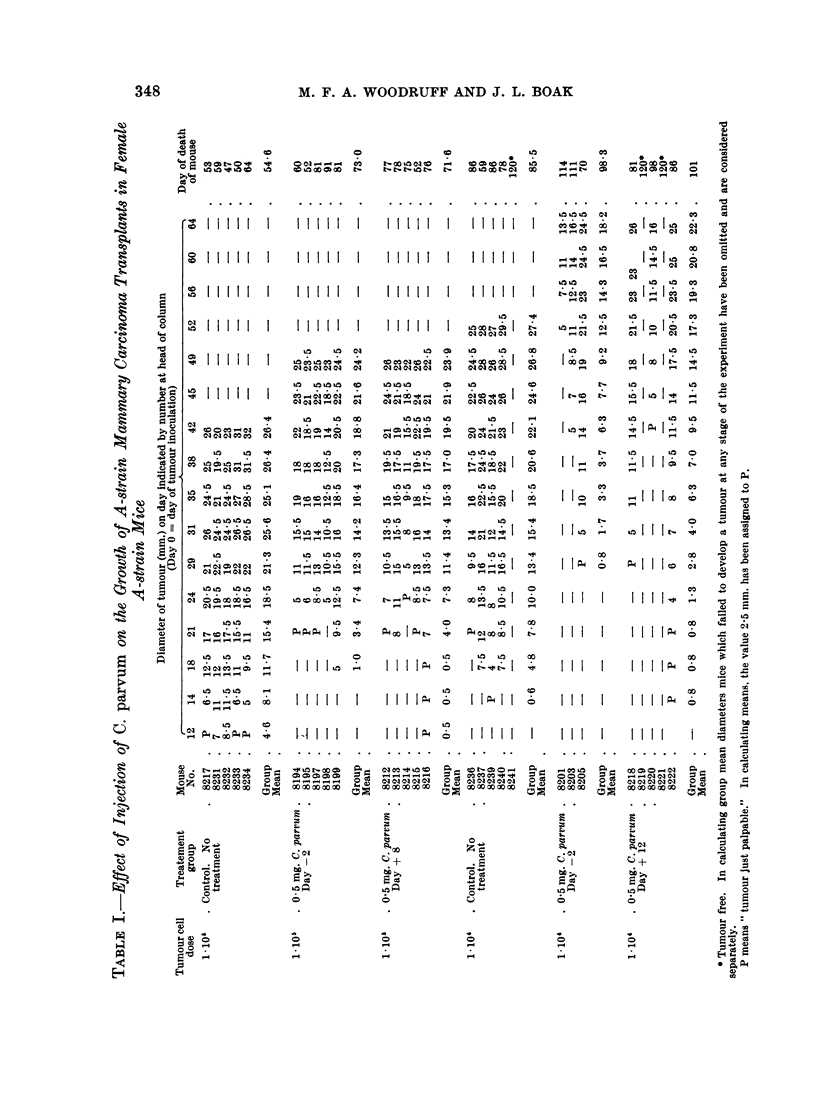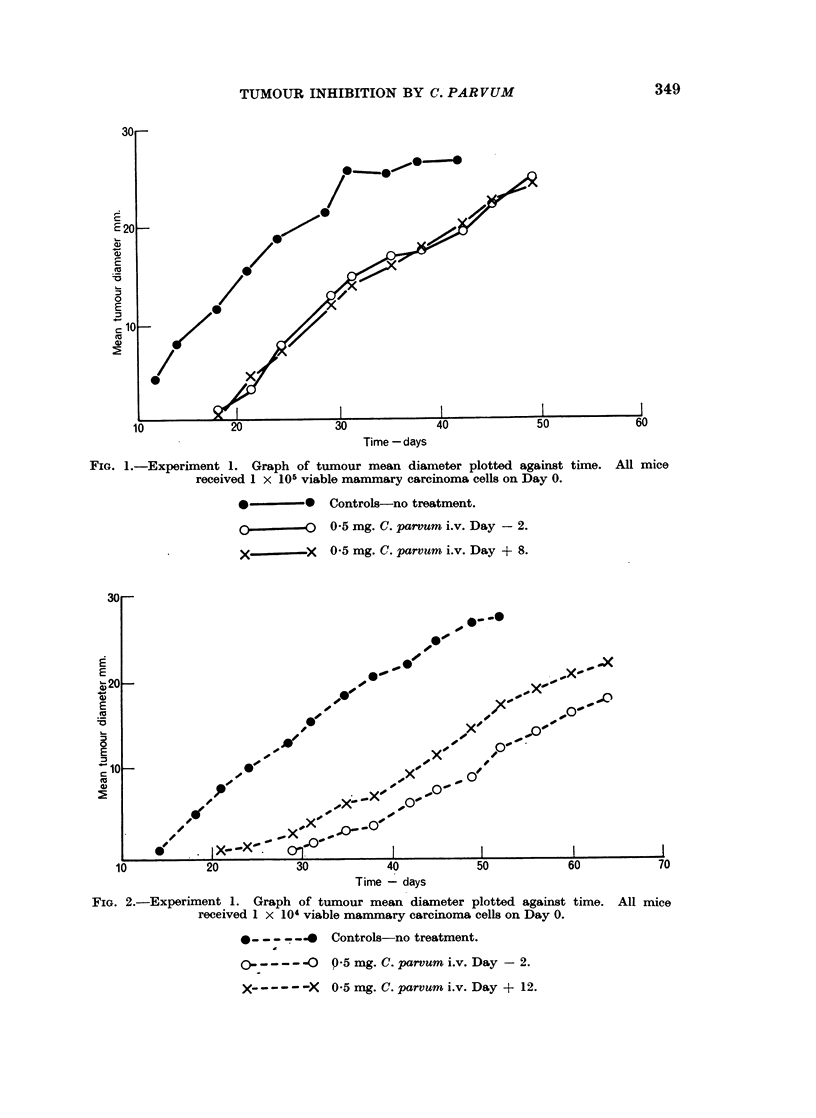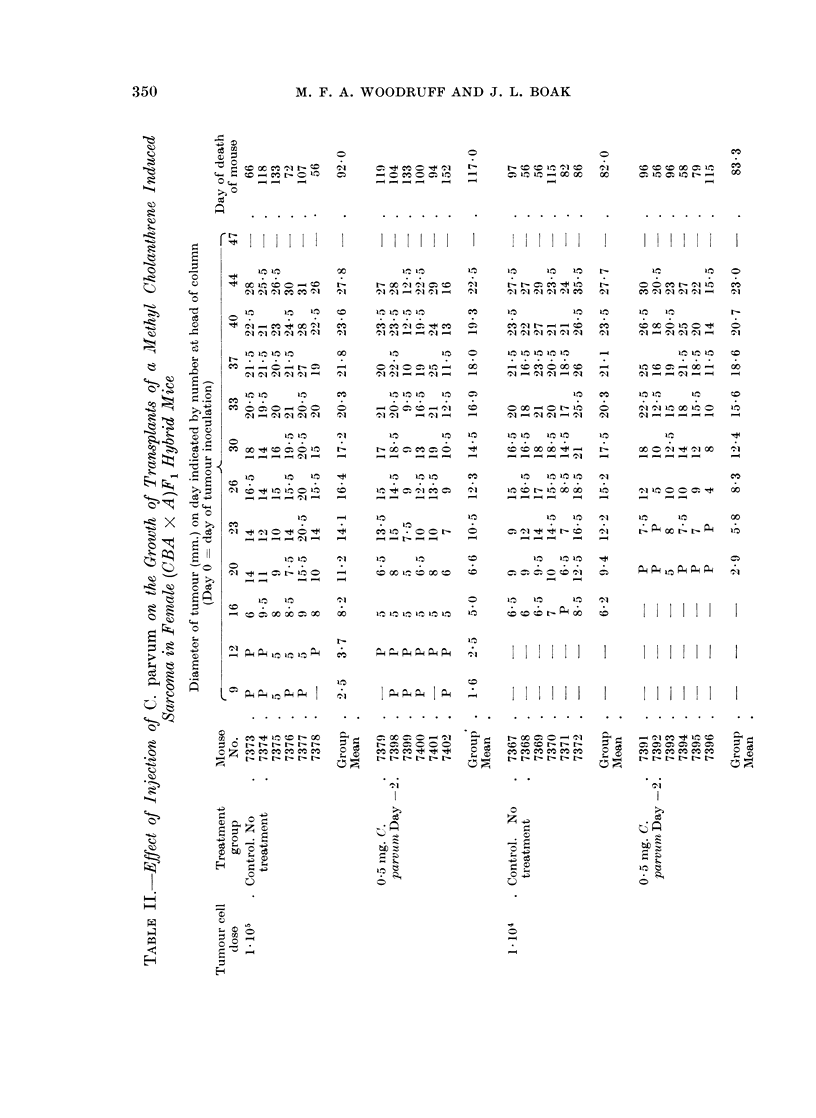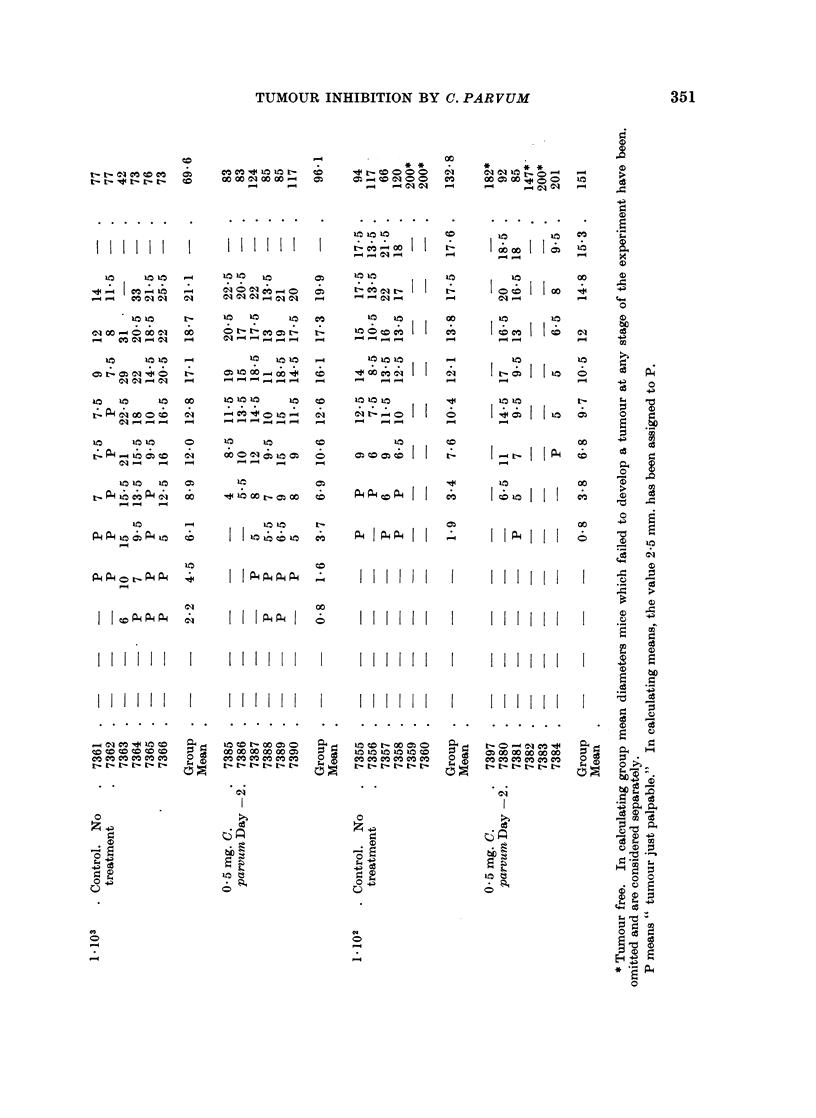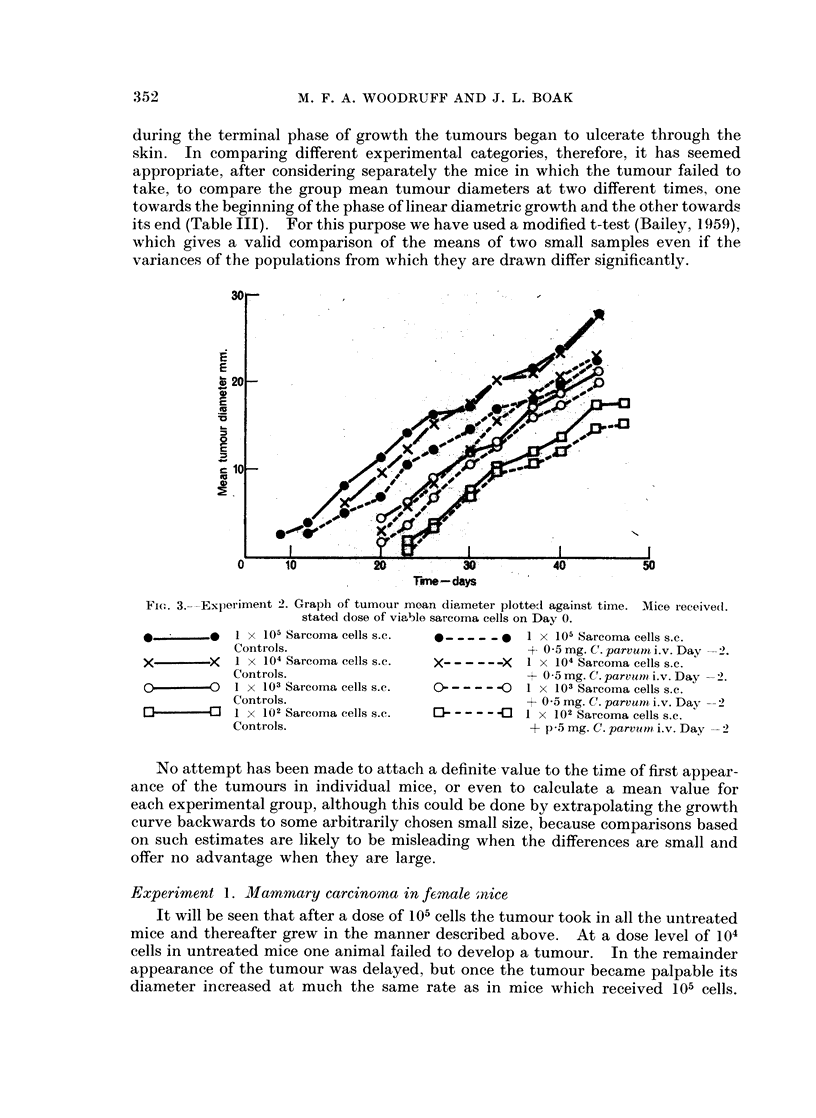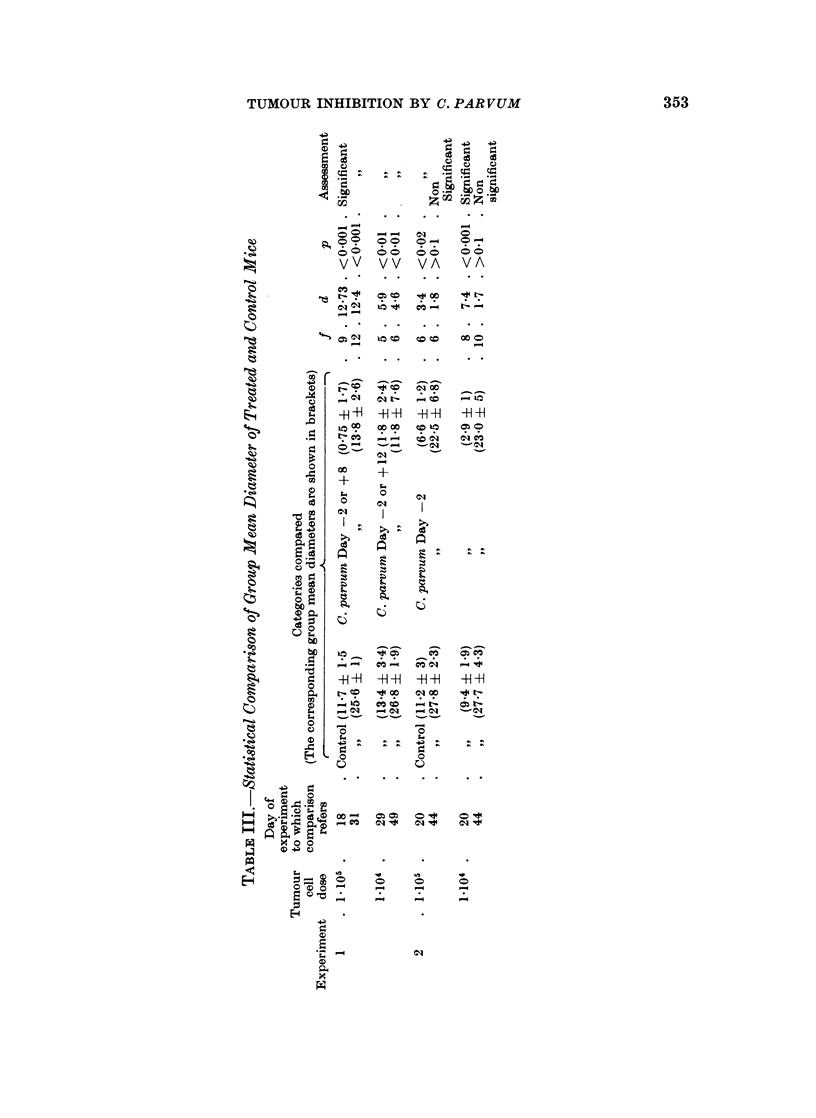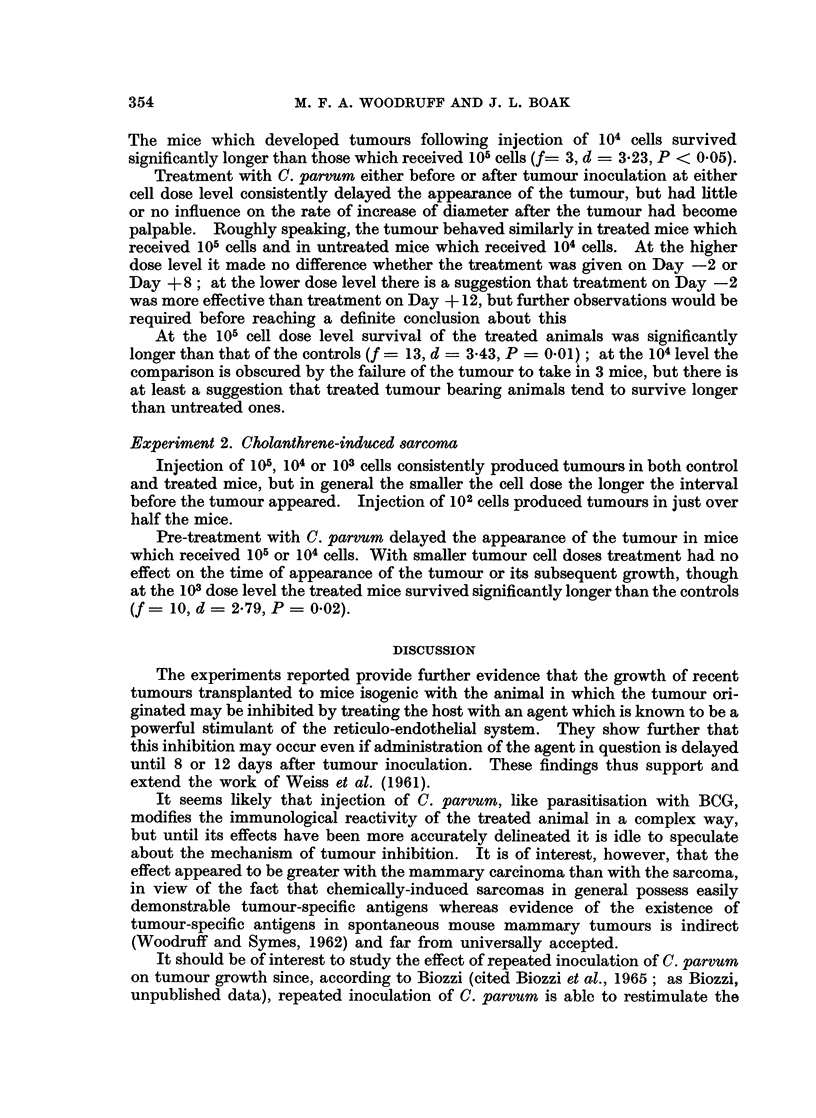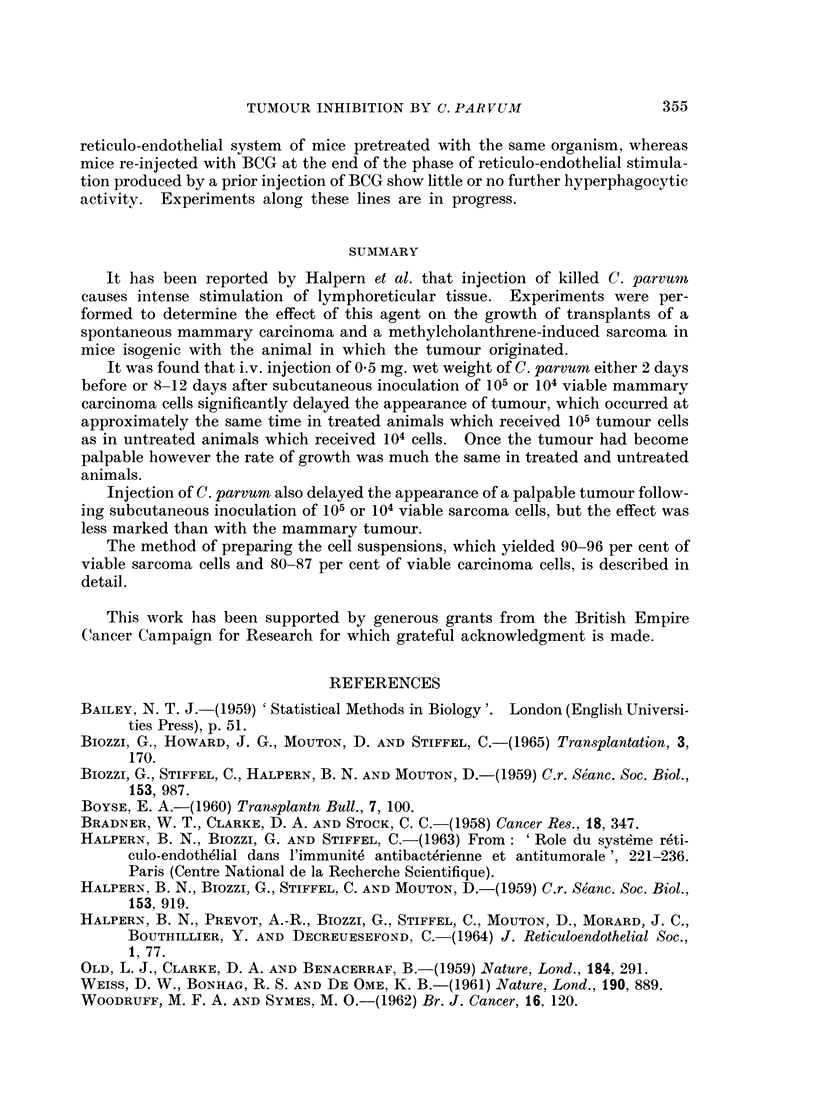# Inhibitory effect of injection of Corynebacterium parvum on the growth of tumour transplants in isogenic hosts.

**DOI:** 10.1038/bjc.1966.42

**Published:** 1966-06

**Authors:** M. F. Woodruff, J. L. Boak


### INHIBITORY EFFECT OF INJECTION OF *CORYNEBACTERIUM PARVUM* ON THE GROWTH OF TUMOUR TRANSPLANTS IN ISOGENIC HOSTS

M. F. A. WOODRUFF AND J. L. BOAK

*From the Department of Surgical Science, University of Edinburgh*

Received for publication January 22, 1966

It has been reported that the growth of some transplanted tumours in mice and rats may be inhibited by prior treatment of the host by various agents which are known to stimulate the phagocytic activity of the reticulo-endothelial system, including zymosan (Bradner, Clarke and Stock, 1958), living *Mycobacterium tuberculosis* (strain BCG) (Biozzi *et al.*, 1959 ; Old, Clarke and Benacerraf, 1959 ; Halpern *et al.*, 1959 ; Halpern, Biozzi and Stiffel, 1963), and a bacterial extract Halpern *et al.*, 1963).

In the experiments cited above the tumours were capable of growing in various strains and were transplanted to animals non-isogeneic with those in which they had originated ; it is, therefore, impossible to assess the significance of the results from the point of view of tumour immunology. This criticism does not apply, however, to the work of Weiss, Bonhag and De Ome (1961), who studied the effect of prior infection with living BCG, injection of phenol-killed BCG, and injection of methanolic extracts of BCG and their residues on the behaviour of five recent mouse tumours transplanted within the strain of origin. They observed various types of effect in the treated animals, including (a) initial retardation of tumour development followed by normal rate of growth, (b) persistent retardation of growth rate, (c) complete prevention of tumour development, (d) initial tumour development followed by regression, (e) inhibition of metastatic spread, and (f) prolonged survival of animals which did develop massive tumours. At certain dose levels some non-living preparations were as effective as, or even more effective than, living BCG, but excessive quantities, even though considerably below the threshold of gross toxicity, sometimes accelerated tumour development. With some preparations treatment three months and one month before tumour transplantation were equally effective.

The present experiments resemble those of Weiss *et al.* (1961) in that the tumours studied were transplanted in mice isogeneic with the mouse in which the particular tumour originated, but differ in the following respects :

1. Injection of killed *Corynebacterium parvum* was used in place of BCG as a means of reticulo-endothelial stimulation. It has been shown by Halpern, Prevot, Biozzi, Stiffel, Mouton, Morard, Bouthillier and Decreuesefond (1964) that single or repeated injection of this agent causes intense stimulation of the lymphoreticular tissues, and recently Biozzi, Howard, Mouton and Stiffel (1965) have reported that it is even more effective than BCG in reducing the mortality from graft-versus-host disease when (C57Bl/6 × C3H/He)$F_1$ hybrid mice are injected with parent line C57Bl/6 spleen cells. The mechanism of the protective effect observed by Biozzi *et al.* is uncertain, but these authors suggest that it is probably due to stimulation of lymphoreticular tissue and consequent increased non-specific resistance to infection.

2. The tumours were transplanted in the form of suspensions containing a measured number of viable cells, and various dose levels were employed.

3. The treatment designed to stimulate reticulo-endothelial activity was given either 2 days before or some time after tumour inoculation.

### MATERIALS AND METHODS

### *General plan of the investigation*

Two experiments were performed. In both, mice received a subcutaneous injection of cells derived from a tumour which had originated in a mouse of the same genetic constitution. Some of the recipients were kept as untreated controls ; others received an intravenous injection of killed *C. parvum* (0·5 mg. wet weight) either 2 days before or 8 or 12 days after the tumour inoculation. Details of the mice and tumours used in each experiment are shown below :

| | | |
|---|---|---|
| Experiment 1. | Host mice : | A-strain females. |
| | Tumour : | First generation transplant of a mammary carcinoma which had originated spontaneously in an A-strain female. |
| Experiment 2. | Host mice : | (CBA $\times$ A)$F_1$ females. |
| | Tumour : | Sarcoma induced by subcutaneous injection of methylcholanthrene in a (CBA $\times$ A)$F_1$ female. |

The mice were inspected twice weekly and if a tumour was present measurements were made with a caliper in its long axis and in a direction perpendicular to this. The arithmetic mean of these two measurements was taken as the mean tumour diameter. Tumours of diameter less than 5 mm. were recorded as palpable (P) ; in calculating group means this has been taken as equivalent to a mean tumour diameter of 2·5 mm.

*Mice.*—The mice were bred in the Department of Surgical Science, University of Edinburgh. The parent strains are checked regularly for uniformity by intrastrain skin grafting.

*Corynebacterium parvum.*—The anaerobic strain 936B was used as described by Halpern *et al.* (1964). Cultures were sterilised by heating to 65° C. for 1 hour and preserved in 0·2 per cent formalin. This material was kindly supplied by Professor Halpern. Injections were made i.v.

*Tumour cell suspensions.*—Tumour cell suspensions were prepared by a modification of the method described by Boyse (1960). The procedure takes only $2\frac{1}{2}$ hours as compared with 4 hours for Boyse's original method, gives a higher yield of viable cells as judged by their failure to stain with trypan blue (approximately $120 \cdot 10^6$ viable cells per g. wet weight tumour with either the mammary carcinoma or the methylcholanthrene induced sarcoma, and less than 20 per cent* non-viable cells), and provides a concentrated suspension in which there is remarkably little

* In 15 successive sarcoma suspensions the proportion of non-viable cells ranged from 4 to 10 per cent. With the mammary carcinoma the proportion of non-viable cells was a little higher, ranging from 13 to 20 per cent in 6 successive preparations.

clumping and which can be diluted as required. In view of these considerable advantages the procedure will be described in detail.

1. The tumour is excised and placed in a sterile Petri dish containing a few ml. of sterile Dulbecco's solution (Oxoid) ; all necrotic looking tissue is cut away and discarded, and the remainder is cut into small pieces (2–4 mm.) with a pair of scalpels (No. 11 blades).

2. The pieces of tumour are transferred to a Melnick flask, washed twice with Dulbecco solution, covered with warmed (37° C.) " Pronase–Dulbecco "* to which has been added 2 drops of Dnase-1 solution (Deoxyribonuclease 1. Sigma London Chemical Co. Ltd.), and incubated in a water bath at 37° C. for 30 minutes. The Dnase-1 solution is prepared at a concentration of 0·2 mg. Dnase-1 per ml. Dulbecco's solution and stored in 2 ml. ampoules at −20° C.

3. The supernatant is poured off and discarded, more Pronase–Dulbecco and Dnase solution is added, and incubation is continued for a further 10 minutes, this time with gentle magnetic stirring.

4. The supernatant (which constitutes the first yield of cell suspension) is decanted through 2-ply sterile gauze into a cold Erlenmeyer flask surrounded by crushed ice.

5. Steps 3 and 4 are repeated twice, and each time the supernatant is added to the cell suspension already harvested.

6. The suspension is strained through stainless steel mesh into cold polypropylene centrifuge tubes. The cells are spun down (310 ***g***. for 10 minutes), washed once in cold 0·15 M NaCl and resuspended in Dulbecco's solution containing a few drops of Dnase solution.

7. The suspension is again strained through stainless steel mesh, counted, and diluted with Dulbecco's solution to give the required cell concentration.

### RESULTS

The experimental protocols are set out in Tables I and II, and the findings are summarised in Fig. 1–3.

In analysing the data it has seemed desirable to examine the effect of both tumour cell dosage and treatment with *C. parvum* on the proportion of animals in which the tumour takes, the time of first appearance of the tumour in animals in which it does take, the pattern of growth after the tumour has become palpable, and the length of survival of the tumour recipient.

Examination of the growth curves for individual mice (which are not reproduced but can be constructed from the tables) shows that with both tumours, as a general rule, there was a period lasting some weeks (referred to hereafter as the phase of linear diametric growth) during which the tumour diameter increased in an approximately linear manner with time, after which the growth rate slackened off. It is not possible from the data to determine the form of the growth curve before the beginning of the phase of linear diametric growth because the tumours were too small to be measured, and the other end of the curve is of little interest because

* Prepared as follows: To 1000 ml. Dulbecco's solution add, in order, 2·5 g. Pronase (Californian Corporation for Biochemical Research, Los Angeles), 500,000 units penicillin, 0·5 g. streptomycin, 0·2 g. neomycin, 5 ml. 0·4 per cent phenol red solution. Adjust pH to 7·2 with 10 per cent NaOH and store unsterilized at −20° C. Thaw in 37° C. water bath the day before use and sterilize by filtration through HA millipore. The sterile material is stored at 4° C. for up to a week and if not used is then discarded.

FIG. 1.—Experiment 1. Graph of tumour mean diameter plotted against time. All mice received 1 × $10^5$ viable mammary carcinoma cells on Day 0.

●———● Controls—no treatment.

○———○ 0·5 mg. *C. parvum* i.v. Day − 2.

×———× 0·5 mg. *C. parvum* i.v. Day + 8.

FIG. 2.—Experiment 1. Graph of tumour mean diameter plotted against time. All mice received 1 × $10^4$ viable mammary carcinoma cells on Day 0.

●- - - - -● Controls—no treatment.

○- - - - -○ 0·5 mg. *C. parvum* i.v. Day − 2.

×- - - - -× 0·5 mg. *C. parvum* i.v. Day + 12.

during the terminal phase of growth the tumours began to ulcerate through the skin. In comparing different experimental categories, therefore, it has seemed appropriate, after considering separately the mice in which the tumour failed to take, to compare the group mean tumour diameters at two different times, one towards the beginning of the phase of linear diametric growth and the other towards its end (Table III). For this purpose we have used a modified t-test (Bailey, 1959), which gives a valid comparison of the means of two small samples even if the variances of the populations from which they are drawn differ significantly.

FIG. 3.—Experiment 2. Graph of tumour mean diameter plotted against time. Mice received stated dose of viable sarcoma cells on Day 0.

| | | | |
|---|---|---|---|
| ●———● | $1 \times 10^5$ Sarcoma cells s.c. Controls. | ●- - - - -● | $1 \times 10^5$ Sarcoma cells s.c. + 0·5 mg. *C. parvum* i.v. Day −2. |
| ×———× | $1 \times 10^4$ Sarcoma cells s.c. Controls. | ×- - - - -× | $1 \times 10^4$ Sarcoma cells s.c. + 0·5 mg. *C. parvum* i.v. Day −2. |
| ○———○ | $1 \times 10^3$ Sarcoma cells s.c. Controls. | ○- - - - -○ | $1 \times 10^3$ Sarcoma cells s.c. + 0·5 mg. *C. parvum* i.v. Day −2 |
| □———□ | $1 \times 10^2$ Sarcoma cells s.c. Controls. | □- - - - -□ | $1 \times 10^2$ Sarcoma cells s.c. + p·5 mg. *C. parvum* i.v. Day −2 |

No attempt has been made to attach a definite value to the time of first appearance of the tumours in individual mice, or even to calculate a mean value for each experimental group, although this could be done by extrapolating the growth curve backwards to some arbitrarily chosen small size, because comparisons based on such estimates are likely to be misleading when the differences are small and offer no advantage when they are large.

*Experiment* 1. *Mammary carcinoma in female mice*

It will be seen that after a dose of $10^5$ cells the tumour took in all the untreated mice and thereafter grew in the manner described above. At a dose level of $10^4$ cells in untreated mice one animal failed to develop a tumour. In the remainder appearance of the tumour was delayed, but once the tumour became palpable its diameter increased at much the same rate as in mice which received $10^5$ cells.

The mice which developed tumours following injection of $10^4$ cells survived significantly longer than those which received $10^5$ cells ($f = 3$, $d = 3{\cdot}23$, $P < 0{\cdot}05$).

Treatment with *C. parvum* either before or after tumour inoculation at either cell dose level consistently delayed the appearance of the tumour, but had little or no influence on the rate of increase of diameter after the tumour had become palpable. Roughly speaking, the tumour behaved similarly in treated mice which received $10^5$ cells and in untreated mice which received $10^4$ cells. At the higher dose level it made no difference whether the treatment was given on Day $-2$ or Day $+8$ ; at the lower dose level there is a suggestion that treatment on Day $-2$ was more effective than treatment on Day $+12$, but further observations would be required before reaching a definite conclusion about this

At the $10^5$ cell dose level survival of the treated animals was significantly longer than that of the controls ($f = 13$, $d = 3{\cdot}43$, $P = 0{\cdot}01$) ; at the $10^4$ level the comparison is obscured by the failure of the tumour to take in 3 mice, but there is at least a suggestion that treated tumour bearing animals tend to survive longer than untreated ones.

*Experiment* 2. *Cholanthrene-induced sarcoma*

Injection of $10^5$, $10^4$ or $10^3$ cells consistently produced tumours in both control and treated mice, but in general the smaller the cell dose the longer the interval before the tumour appeared. Injection of $10^2$ cells produced tumours in just over half the mice.

Pre-treatment with *C. parvum* delayed the appearance of the tumour in mice which received $10^5$ or $10^4$ cells. With smaller tumour cell doses treatment had no effect on the time of appearance of the tumour or its subsequent growth, though at the $10^3$ dose level the treated mice survived significantly longer than the controls ($f = 10$, $d = 2{\cdot}79$, $P = 0{\cdot}02$).

### DISCUSSION

The experiments reported provide further evidence that the growth of recent tumours transplanted to mice isogenic with the animal in which the tumour originated may be inhibited by treating the host with an agent which is known to be a powerful stimulant of the reticulo-endothelial system. They show further that this inhibition may occur even if administration of the agent in question is delayed until 8 or 12 days after tumour inoculation. These findings thus support and extend the work of Weiss *et al.* (1961).

It seems likely that injection of *C. parvum*, like parasitisation with BCG, modifies the immunological reactivity of the treated animal in a complex way, but until its effects have been more accurately delineated it is idle to speculate about the mechanism of tumour inhibition. It is of interest, however, that the effect appeared to be greater with the mammary carcinoma than with the sarcoma, in view of the fact that chemically-induced sarcomas in general possess easily demonstrable tumour-specific antigens whereas evidence of the existence of tumour-specific antigens in spontaneous mouse mammary tumours is indirect (Woodruff and Symes, 1962) and far from universally accepted.

It should be of interest to study the effect of repeated inoculation of *C. parvum* on tumour growth since, according to Biozzi (cited Biozzi *et al.*, 1965 ; as Biozzi, unpublished data), repeated inoculation of *C. parvum* is able to restimulate the reticulo-endothelial system of mice pretreated with the same organism, whereas mice re-injected with BCG at the end of the phase of reticulo-endothelial stimulation produced by a prior injection of BCG show little or no further hyperphagocytic activity. Experiments along these lines are in progress.

### SUMMARY

It has been reported by Halpern *et al.* that injection of killed *C. parvum* causes intense stimulation of lymphoreticular tissue. Experiments were performed to determine the effect of this agent on the growth of transplants of a spontaneous mammary carcinoma and a methylcholanthrene-induced sarcoma in mice isogenic with the animal in which the tumour originated.

It was found that i.v. injection of 0·5 mg. wet weight of *C. parvum* either 2 days before or 8–12 days after subcutaneous inoculation of $10^5$ or $10^4$ viable mammary carcinoma cells significantly delayed the appearance of tumour, which occurred at approximately the same time in treated animals which received $10^5$ tumour cells as in untreated animals which received $10^4$ cells. Once the tumour had become palpable however the rate of growth was much the same in treated and untreated animals.

Injection of *C. parvum* also delayed the appearance of a palpable tumour following subcutaneous inoculation of $10^5$ or $10^4$ viable sarcoma cells, but the effect was less marked than with the mammary tumour.

The method of preparing the cell suspensions, which yielded 90–96 per cent of viable sarcoma cells and 80–87 per cent of viable carcinoma cells, is described in detail.

This work has been supported by generous grants from the British Empire Cancer Campaign for Research for which grateful acknowledgment is made.

### REFERENCES

BAILEY, N. T. J.—(1959) ' Statistical Methods in Biology '. London (English Universities Press), p. 51.

BIOZZI, G., HOWARD, J. G., MOUTON, D. AND STIFFEL, C.—(1965) *Transplantation*, **3**, 170.

BIOZZI, G., STIFFEL, C., HALPERN, B. N. AND MOUTON, D.—(1959) *C.r. Séanc. Soc. Biol.*, **153**, 987.

BOYSE, E. A.—(1960) *Transplantn Bull.*, **7**, 100.

BRADNER, W. T., CLARKE, D. A. AND STOCK, C. C.—(1958) *Cancer Res.*, **18**, 347.

HALPERN, B. N., BIOZZI, G. AND STIFFEL, C.—(1963) From : ' Role du systéme réticulo-endothélial dans l'immunité antibactérienne et antitumorale ', 221–236. Paris (Centre National de la Recherche Scientifique).

HALPERN, B. N., BIOZZI, G., STIFFEL, C. AND MOUTON, D.—(1959) *C.r. Séanc. Soc. Biol.*, **153**, 919.

HALPERN, B. N., PREVOT, A.-R., BIOZZI, G., STIFFEL, C., MOUTON, D., MORARD, J. C., BOUTHILLIER, Y. AND DECREUESEFOND, C.—(1964) *J. Reticuloendothelial Soc.*, **1**, 77.

OLD, L. J., CLARKE, D. A. AND BENACERRAF, B.—(1959) *Nature, Lond.*, **184**, 291.

WEISS, D. W., BONHAG, R. S. AND DE OME, K. B.—(1961) *Nature, Lond.*, **190**, 889.

WOODRUFF, M. F. A. AND SYMES, M. O.—(1962) *Br. J. Cancer*, **16**, 120.